# Cancer-associated fibroblasts and metabolic reprogramming predict pathologic response to neoadjuvant PD-1 blockade in resected non-small cell lung cancer

**DOI:** 10.1007/s13402-025-01067-4

**Published:** 2025-05-13

**Authors:** Jiaqi Zhao, Maolin Liu, Chongmei Zhu, Zhuolin Li, Zuhui Liu, Dilimulati Abulizi, Siqing Liu, Xin Wang, Haoxian Yang, Xue Hou

**Affiliations:** 1 Department of Medical Oncology, State Key Laboratory of Oncology in South China, Collaborative Innovation Center for Cancer Medicine, Sun Yat-Sen University Cancer Center, No. 651, Dongfeng East Road, Guangzhou City, Guangdong Province 510060 PR China; 2 Department of Pathology, State Key Laboratory of Oncology in South China, Collaborative Innovation Center for Cancer Medicine, Sun Yat-sen University Cancer Center, Guangzhou, PR China; 3 Guangzhou BioScript Biotechnology Co., Ltd, Guangzhou, PR China; 4 The Department of Breast Disease, The Fifth Affiliated Hospital, Sun Yat-sen University, Zhuhai, PR China; 5 Department of Thoracic Surgery, State Key Laboratory of Oncology in South China, Collaborative Innovation Center for Cancer Medicine, Sun Yat-Sen University Cancer Center, No. 651, Dongfeng East Road, Guangzhou City, Guangdong Province 510060 PR China

**Keywords:** Lung cancer, Neoadjuvant immunotherapy, Cancer-associated fibroblasts, Metabolic reprogramming

## Abstract

**Purpose:**

Immunotherapy has transformed the neoadjuvant treatment landscape for patients with resectable locally advanced non-small cell lung cancer (NSCLC). However, a population of patients cannot obtain major pathologic response (MPR) and thus benefit less from neoadjuvant immunotherapy, highlighting the need to uncover the underlying mechanisms driving resistance to immunotherapy.

**Methods:**

Two published single-cell RNA sequencing (scRNA-seq) datasets were used to analyze the subsets of cancer-associated fibroblasts (CAFs) and T cells and functional alterations after neoadjuvant immunotherapy. The stromal signature predicting ICI response was identified and validated using our local cohort with stage III NSCLC receiving neoadjuvant immunotherapy and other 4 public ICI transcriptomic cohorts.

**Results:**

Non-MPR tumors showed higher enrichment of CAFs and increased extracellular matrix deposition than MPR tumors, as suggested by bioinformatic analysis. Further, CAF-mediated immune suppression may involve reciprocal interactions with T cells in addition to a physical barrier mechanism. In contrast, MPR tumors demonstrated therapy-induced activation of memory CD8^+^ T cells into an effector phenotype. Additionally, neoadjuvant immunotherapy resulted in expansion of precursor exhausted T (Texp) cells, which were remodeled into an anti-tumor phenotype. Notably, we identified metabolic heterogeneity within distinct T cell clusters during immunotherapy. Methionine recycling emerged as a predictive factor for T-cell differentiation and a favorable pathological response. The stromal signature was associated with ICI response, and this association was validated in five independent ICI transcriptomic cohorts.

**Conclusion:**

These discoveries underscore the distinct tumor microenvironments in MPR and non-MPR patients and may elucidate resistance mechanisms to immunotherapy in NSCLC.

**Supplementary Information:**

The online version contains supplementary material available at 10.1007/s13402-025-01067-4.

## Introduction

Cancer-related deaths are most frequently attributed to lung cancer globally, predominantly in the form of non-small cell lung cancer (NSCLC) [1]. Stage III NSCLC is a group of heterogeneous diseases with different prognosis and needs multidisciplinary treatment strategies. For potentially resectable stage III NSCLC, neoadjuvant platinum-based chemotherapy increased the resection rate with moderate survival benefit [2]. Along with revolutionary advances of immune checkpoint blockade (ICB) in metastatic NSCLC, neoadjuvant immunotherapy in resectable NSCLC greatly improved surgery outcome and survival as well, and gradually established its solid status [3–5]. Major pathologic response (MPR) was proved to be an alternative indicator of survival benefit in neoadjuvant immunotherapy of NSCLC; nevertheless, a significant proportion of patients fail to achieve MPR with neoadjuvant immunotherapy. Currently, MPR was assessed based on the administration of surgery. Consequently, there is a need to develop additional biomarkers that are indicative of the pathological response to neoadjuvant immunotherapy.

The levels of PD-L1 expression and the tumor mutational burden (TMB), as the most widely used biomarkers, remain controversial for predicting clinical benefit of neoadjuvant immunotherapy [3, 4, 6, 7]. The tumor microenvironment (TME) represents another aspect of potential biomarkers for immunotherapy efficacy and has been explored. Reuss and colleagues [7] showed that abundant CD8^+^ T cells were observed in patients with pathologic complete response (pCR) to neoadjuvant nivolumab plus ipilimumab (NCT02259621). In the NADIM study (NCT03081689) [8], *IFNG*, *GZMB*, *NKG7*, and M1 macrophages were positively correlated with pCR using bulk RNA sequencing. More recently, two distinct studies leveraged single-cell RNA sequencing (scRNA-seq) to meticulously analyze a variety of immune cell subtypes within the TME to explore the response of neoadjuvant immunotherapy [9, 10]. However, the focus of these studies was primarily on immune cells, with the stromal elements of the TME not yet receiving adequate exploration.

Currently, there is a paucity of data regarding whether cancer associated fibroblasts (CAFs) are capable of triggering immunotherapy resistance in neoadjuvant chemoimmunotherapy and how treatment reprograms the environmental signals, leading to altered functional phenotypes of T cells. Thus, further investigations are needed to explore the potential of new biomarkers to guide patient selection in neoadjuvant immunotherapies.

In this study, we revealed the CAFs and metabolic flux and their correlation with the effectiveness of immunotherapy using 2 scRNA-seq datasets and 5 independent cohorts, including public cohorts and our local cohort with stage III NSCLC receiving neoadjuvant immunotherapy. Our findings uncovered the potential of the stromal signature for predicting the pathologic response to neoadjuvant PD-1 blockade in patients with locally advanced resectable NSCLC.

## Materials and methods

### scRNA-seq datasets analysis

Two lung carcinoma datasets (http://lungcancer.chenlulab.com(11), GSE176022 [9]) were analyzed in terms of the proportions of single cells and function alterations after neoadjuvant immunotherapy. The analysis was performed following the Seurat single-cell analysis standard workflow [11]. Initially, each dataset was utilized to create a Seurat object. To ensure data quality, cells with fewer than 300 measured genes or more than 20% mitochondrial contamination were deemed as low-quality cells and subsequently excluded. Additionally, cells with over 5000 measured genes were identified as potential doublets and eliminated from the datasets. Following the filtration step, the merged object was normalized using the “NormalizeData” function with the method set to “LogNormalize” and a scale factor of 10,000. Subsequently, the 3000 most variable genes were identified, and their expression levels were scaled prior to performing principal component analysis (PCA) within the variable gene space. Harmony (version 1.0) [12] was applied to correct batch effects and integrate the merged object. Cell clustering based on cell type (including epithelial cells, CD8^+^ T cells, CD4^+^ T cells, stromal cells, macrophages, and dentritic cells) was carried out using a graph-based clustering method. Unsupervised clustering analysis was performed using the top principal components, and the results were visualized using Uniform Manifold Approximation and Projection (UMAP) for dimensionality reduction. The Seurat package was employed to annotate and group the clusters by cell type. This was achieved by manually inspecting differentially expressed genes in each cluster using the MAST method [13], with canonical marker genes from the literature used as references. The selected markers were then visualized on UMAP coordinates as gene expression density using the R package Nebulosa [14]. The Monocle 2 package [15] was employed to provide pseudotime cell distributions for each cell type. To infer cell-cell interactions between different cell types, the CellPhoneDB (version 2.1.5) [16] method was utilized. This method determines the potential interaction strength between two cell subsets based on gene expression levels and assesses significance through permutation tests (1000 iterations). Enriched ligand-receptor interactions between cell subsets were calculated using these permutation tests.

### Single Cell Flux Estimation Analysis (scFEA)

To estimate the cell-wise metabolic flux rates against the whole human metabolic map, the python package scFEA [17] version 1.2 was employed. This analysis was performed using publicly available scRNA-seq data from the dataset GSE176022. The Mann-Whitney test was applied to assess the statistical significance of the differences in metabolic flux between cell groups.

### Validation cohorts

To translate the previous in silico findings into clinical samples, we performed analysis of 20 stage III NSCLC patients in a prospective study at Sun Yat-Sen University Cancer Center (SYSUCC, Guangzhou, China). This study was approved by the Institutional Review Board of SYSUCC [approval no. B2017-001-Y01], and written informed consent was obtained from all patients. Main inclusion criteria included following: (1) NSCLC newly diagnosed by histopathology; (2) clinical stage III NSCLC and potentially resectable; (3) neoadjuvant PD-1 inhibitors plus platinum-based chemotherapy followed by complete resection were administered; (4) pre- and post-treatment tumor tissues were available; (5) tumors were confirmed as negative for known driver gene mutations, including *KRAS*, *EGFR*, and *ALK*.

Between June 2018 and October 2021, a total of 20 patients receiving neoadjuvant PD-1 inhibitor plus chemotherapy were enrolled in the study. Pre-treatment primary tumor tissues were obtained through percutaneous pulmonary biopsy, bronchoscopy, or endobronchial ultrasound-guided biopsy before drug administration. Patients received one to three cycles of neoadjuvant immunotherapy-chemotherapy combination before surgery. Post-treatment tumor tissues were collected immediately after surgical resection, and pathological response was assessed as the percentage of residual viable tumor (%RVT) at the primary tumor identified on routine hematoxylin and eosin staining [18]. Tumors with no more than 10% viable tumor cells were considered to exhibit major pathologic responses (MPR). Forty tumor samples of 20 patients enrolled were subjected to RNA extraction and sequencing. Thirty-three of 40 samples had a good sequencing quality and presented for further analysis: 17 of them were obtained at diagnosis and 16 after radical surgery. Thirteen of them were paired samples (pre-treatment and post-treatment) in which RNA sequencing was done at both timepoints.

The bulk RNA-seq datasets GSE207422 [10], GSE135222 [19, 20], the SU2C-MARK Cohort [21], and a metastatic melanoma cohort [22] were downloaded for validation.

### RNA extraction

Bulk RNA was extracted from formalin-fixed paraffin embedded (FFPE) tissues utilizing Trizol reagent and the RNeasy MinElute Cleanup Kit (Cat. No. 74204). The purity of the RNA was determined with the kaiaoK5500^®^Spectrophotometer (Kaiao, Beijing, China). Acceptance criteria for RNA quality were an optical density ratio (OD260/OD280) within the range of 1.8 to 2.0. The integrity and concentration of the RNA samples were evaluated using the RNA Nano 6000 Assay Kit on the Bioanalyzer 2100 system (Agilent Technologies, CA, USA). According to the manufacturer’s protocol, mRNA libraries were prepared using the NEB Next^®^ Ultra™ RNA Library Prep Kit for Illumina^®^ (#E7530L, NEB, USA). The constructed RNA-seq libraries were sequenced using the DNBSEQ-T7RS sequencer (MGI Tech).

### Bulk RNA sequencing data analysis

The sequencing reads containing adaptor sequences and low-quality reads were removed to obtain high-quality paired-end reads. These reads were aligned to the human genome (hg19) using HISAT (v2.0.4) Transcript assembly was performed using StringTie (v1.2.3). Differential-expressed genes (DEGs) between groups were assessed using the DESeq2 package (|fold change| ≥ 2 and adjusted p value < 0.05).

### Functional and pathway enrichment analysis

Functional and pathway enrichment was done using single-sample gene-set enrichment analysis (ssGSEA) algorithm [23]. Gene sets were downloaded from the IOBR (https://github.com/IOBR/IOBR) [24], MatrisomeDB [25], KEGG database (http://www.kegg.jp), and TIP (Tracking Tumor Immunophenotype) (http://biocc.hrbmu.edu.cn/TIP/) [26]. Significantly disturbed pathways were identified with Benjamini-Hochberg-corrected P value of ≤ 0.05. To estimate the proportions of the immune cell types in the tumor microenvironment, the analytical tool xCell was used [27].

### Histologic staining

Multiplex immunofluorescence staining was performed using PANO 7-plex IHC kit (cat#0004100100, Panovue, Beijing, China). The following primary antibodies were used: anti-α-SMA (Abcam, ab5694); anti-CD8A (CST, CST70306); and anti-panCK (Sigma, C2562) to mark CAFs, immune cells, and tumor epithelial cells, respectively. Thereafter, slides were incubated with secondary antibodies, followed by tyramide signal amplification (TSA). Next, slides were subjected to microwave heat-treated antigen retrieval after each TSA operation. Finally, nuclei were stained with DAPI (Sigma-Aldrich, D9542) after all antigens had been labeled. To obtain multispectral images, the stained slides were scanned using an Olympus VS200 (Olympus Germany), in conjunction with Olympus UPLXAPO 20x objective lens. The quantitative analysis was performed using QuPath software.

To assess collagen deposition in the tumor tissue, Masson’s trichrome staining was performed by pathologist Zhu CM.

### Establishment of the prognostic risk score

In the study, the random survival forest (RSF) model from the “randomForestSRC” R package (version 3.2.2) was utilized to filter candidate genes associated with both survival and ECM. The RSF algorithm ranked each gene based on its importance, and the top 26 most important genes were selected for constructing the risk score model in the analysis. To assess the association between the selected gene signature and patient survival, the patients were divided into high and low signature groups using the median value of the risk score. Kaplan-Meier survival curves with the cumulative number of events table and log-rank test were plotted by survminer (version 0.4.9) and survival (version 3.5.7) R package.

### Statistical analysis

The differences across groups were calculated by non-parametric MannWhitney U test, Wilcoxon rank-sum test, Pearson Correlation test, or one-way ANOVA followed by Tukey’s post hoc test for multiple comparisons as indicated. For survival analysis, a log-rank test was used to compare curves between groups. All tests were two-sided. Statistical analyses were performed in the R (v. 4.3.1) or GraphPad Prism (v. 9.0; GraphPad Software, La Jolla, CA, USA) software.

## Results

### The response to neoadjuvant immunotherapy is linked to the ratios of CD8^**+**^ T cells and cancer-associated fibroblasts

**Fig. 1:**
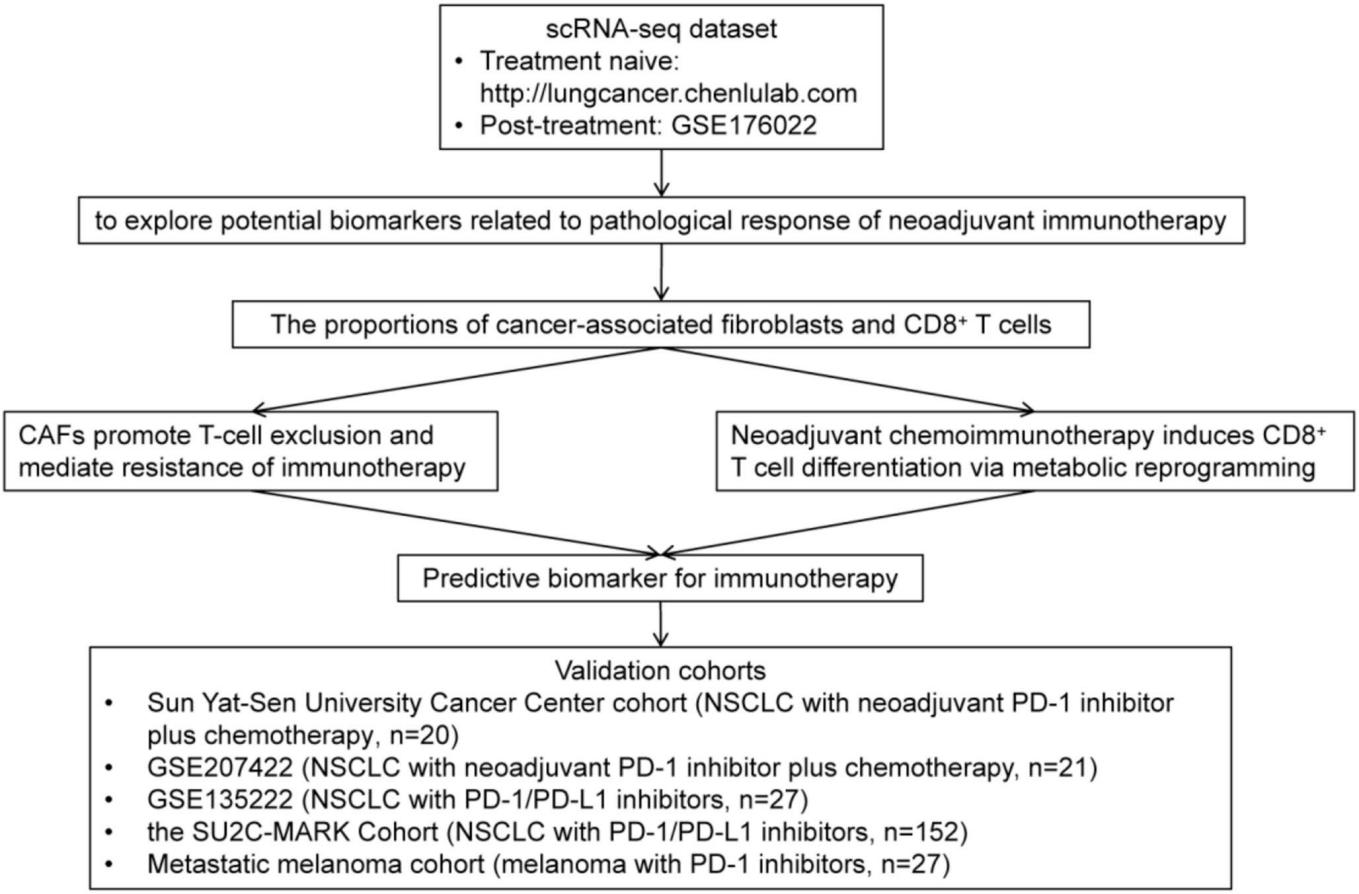
Scheme of the overall study design

**Fig. 2:**
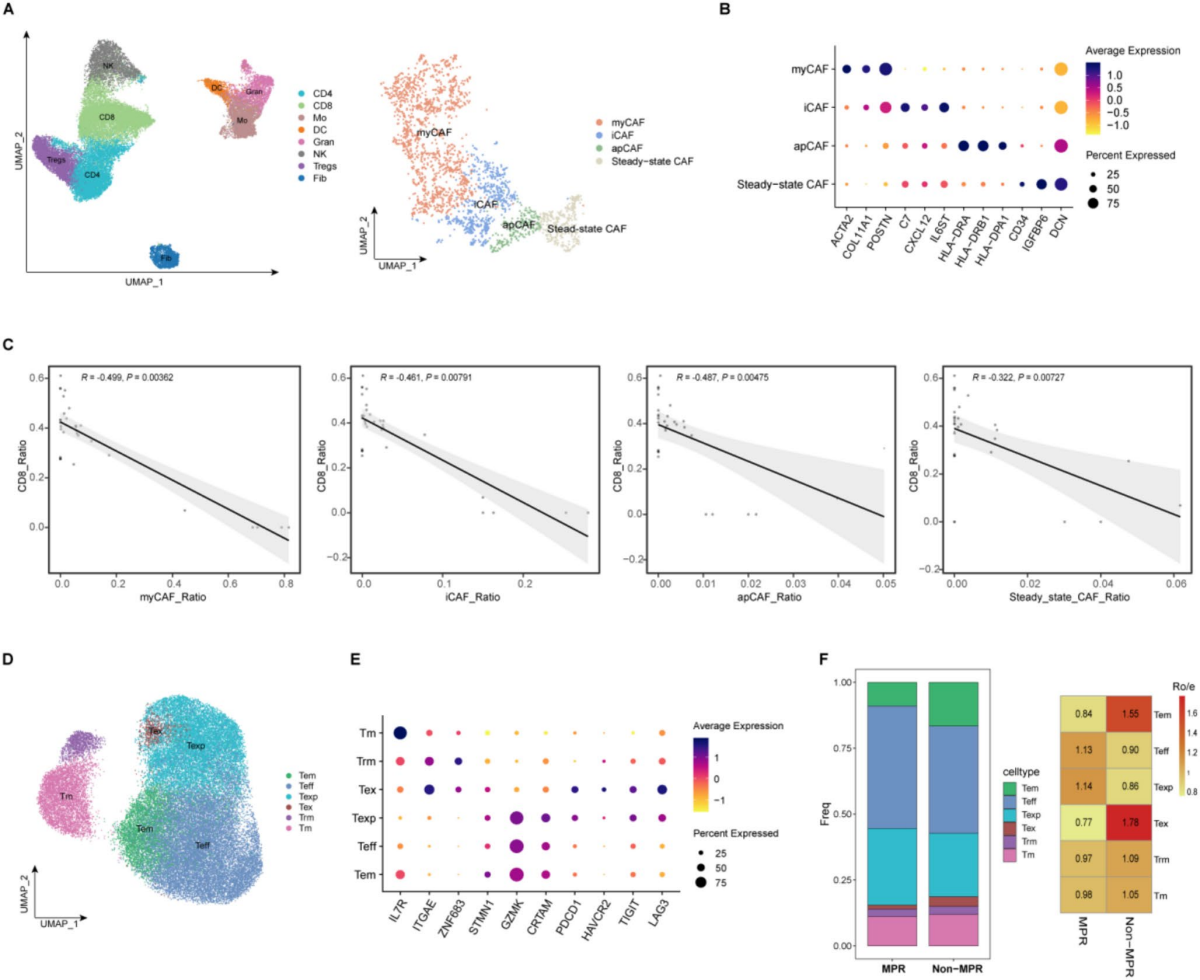
(**A**) The proportions of cancer-associated fibroblasts and CD8^+^ T cells. Uniform manifold approximation and projection (UMAP) plot of all cells (left) and fibroblasts (right) colored by major cell types according to canonical markers. (Data source: http://lungcancer.chenlulab.com).(**B**) Heatmap showing the expression levels of selected marker genes in different CAF cell groups from A. (**C**) Estimating the correlation between the compositional ratios of CAF subtypes and CD8^+^ T cell populations (derived from **A**). The corresponding Pearson correlation values are shown. (**D**) UMAP plot of CD8^+^ T cells colored by clusters from scRNA data (GSE176022). (**E**) Heatmap of normalized expression of canonical CD8^+^ T cell marker genes among clusters. (**F**) (Left) Bar plots indicating the proportion of major cell lineages in each group from **B**. (Right) Relative overrepresentation (Ro/e) analysis of cluster distributions among different groups. Significance was determined by Pearson correlation test (**C**)

The flowchart of this study was displayed in Fig. 1. Given the reported immunoregulatory properties of CAF [28–30], we aimed to understand the function of different subsets of CAFs within the TME and their impact on treatment response in this study. A scRNA-seq dataset from Zhang’s study [31] was used to re-clustered CAF into four distinct subclusters (Fig. 2A). DEG analysis revealed subtype-specific molecular signatures (Fig. 2B and Table S1): the first subcluster was annotated as myofibroblast-like CAFs (myCAFs), characterized by elevated expression of extracellular matrix (ECM) remodeling genes (*ACTA2* [α-smooth muscle actin, α-SMA], *COL11A1*, *POSTN*), consistent with their role in stromal stiffening and mechanical force transduction. The second subcluster, inflammatory CAFs (iCAFs), exhibited a pro-inflammatory phenotype marked by cytokine signaling components (*IL6ST*, *C7*) and chemokines (*CXCL12*), suggesting involvement in immune cell recruitment and immunosuppressive niche formation. The third subcluster displayed antigen-presenting features (apCAFs), including enrichment of MHC class II molecules (*HLA-DRA*, *HLA-DRB1*, *HLA-DPA1*), indicative of potential immunomodulatory crosstalk. Finally, the fourth subcluster, designated steady-state CAFs, retained quiescent fibroblast properties with baseline stromal markers (*DCN*, *IGFBP6*) and minimal inflammatory activity, likely representing a homeostatic population maintaining tissue architecture. Interestingly, we observed a significant inverse correlation between the abundance of all CAF subpopulations and CD8^+^ T cell infiltration levels (Fig. 2C), suggesting potential immunosuppressive crosstalk within the tumor microenvironment.

To delve deeper into the characteristics of the TME as it undergoes changes in response to therapy, we performed a re-clustering analysis of CD8^+^ T cells using publicly available scRNA-seq data from post-treatment patients with NSCLC, as obtained from the dataset GSE176022. This analysis identified six clusters of CD8^+^ T cells, which include memory T cells (Tm), effector memory T cells (Tem), tissue-resident memory T cells (Trm), effector T cells (Teff), exhausted T cells (Tex), and precursor exhausted T cells (Texp), each with its unique signature genes (Fig. 2D-E, and Fig. S1). Further examination revealed a sizable increase in expanded Texp cells in the group of patients exhibiting MPR as opposed to those in the non-MPR group (Fig. 2F). This finding corroborates the hypothesis that Texp cells play a pivotal role in the efficacy of ICB therapy [32].

### Cancer-associated fibroblasts promote T-cell exclusion and mediate resistance of immunotherapy

**Fig. 3:**
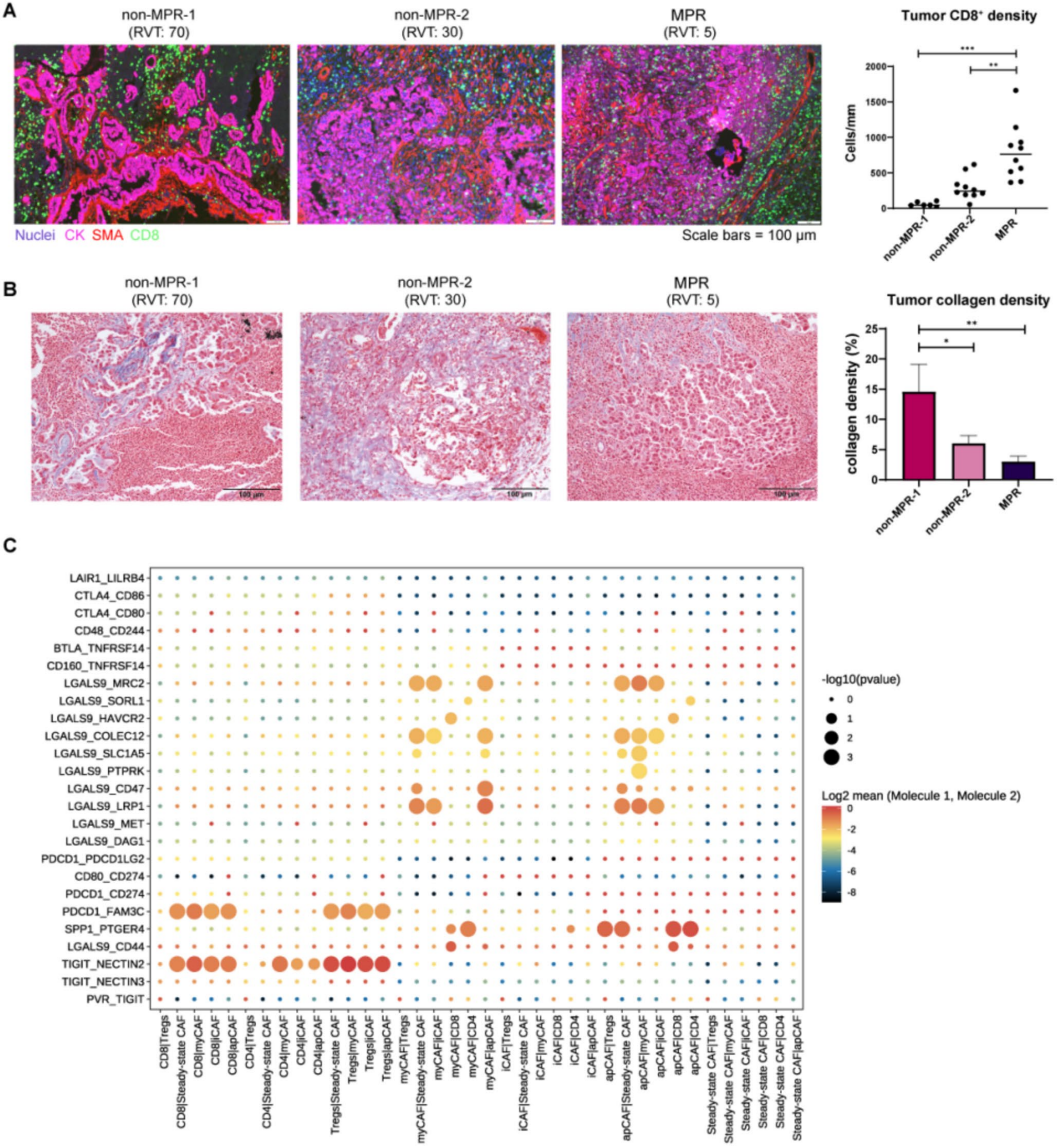
Cancer-associated fibroblasts promote T-cell exclusion and mediate treatment resistance of chemoimmunotherapy. In situ multiplex immunofluorescence (mIF) analysis of CAF and CD8^+^ T cells in MPR and non-MPR patients from pre-treatment biopsies. Representative images (left) and quantification of the ratio of CD8^+^ cells per mm^2^ in the tumor (right) are shown. Masson’s trichrome (MT) staining for collagen density in MPR and non-MPR patients from pre-treatment biopsies. Representative images (left) and quantification of the collagen density in the tumor (right) are shown

To validate our in silico findings within actual clinical specimens, we conducted an analysis of CD8 and α-SMA expression, as detected by multiplex immunofluorescence staining, within a local cohort of stage III NSCLC patients who received neoadjuvant immunotherapy. SMA^+^ CAFs were identified as pervasive within the tumor stroma, often manifesting as organized cell layers that encircled tumor nests in a subset of the malignancies, consistent with a higher ratio of infiltrating CD8^+^ cells in MPR tumors compared to non-MPR tumors (Fig. 3A). Furthermore, in non-MPR tumors, a pronounced alignment of collagen fibers was detected through Masson’s trichrome staining (Fig. 3B). This observation suggests that the dense matrix fibers might play a functional role in impeding the interactions between T cells and the cancer cells, potentially influencing the efficacy of T cell-mediated immune responses within the TME.

To quantify the possible adverse impact of CAFs on CD8^+^ T cells, we next sought to investigate how spatially positioning of CAF interacts with immune cells within the TME. CellPhoneDB analysis revealed enrichment of interactions between all CAF subtypes and CD8^+^ T cells as well as Treg cells, including *TIGIT-NECTIN2* and *PDCD1-FAM3C* interactions (Fig. 3C). Of particular interest was the finding that apCAFs may orchestrate immunosuppressive crosstalk with multiple T cell populations (Tregs, CD8^+^ T, and CD4^+^ T cells) through *SPP1*-*PTGER4* signaling, suggesting a potential mechanism for apCAF-mediated immune evasion. Additionally, we observed significant enrichment of *LGALS9-CD44* and *LGALS9-HAVCR2* interactions between myCAFs and CD8^+^ T cells (Fig. 3C). This finding was supported by *LGALS9* (the gene encoding galectin-9) expression patterns showing predominant localization in myCAFs and iCAFs (Fig. S2). Collectively, these results highlight a potential multifaceted role for CAFs in shaping an immunosuppressive tumor microenvironment through distinct subtype-specific interactions with cytotoxic and regulatory T cell populations.

### Neoadjuvant chemoimmunotherapy induces CD8^+^ T cell differentiation via metabolic reprogramming

**Fig. 4:**
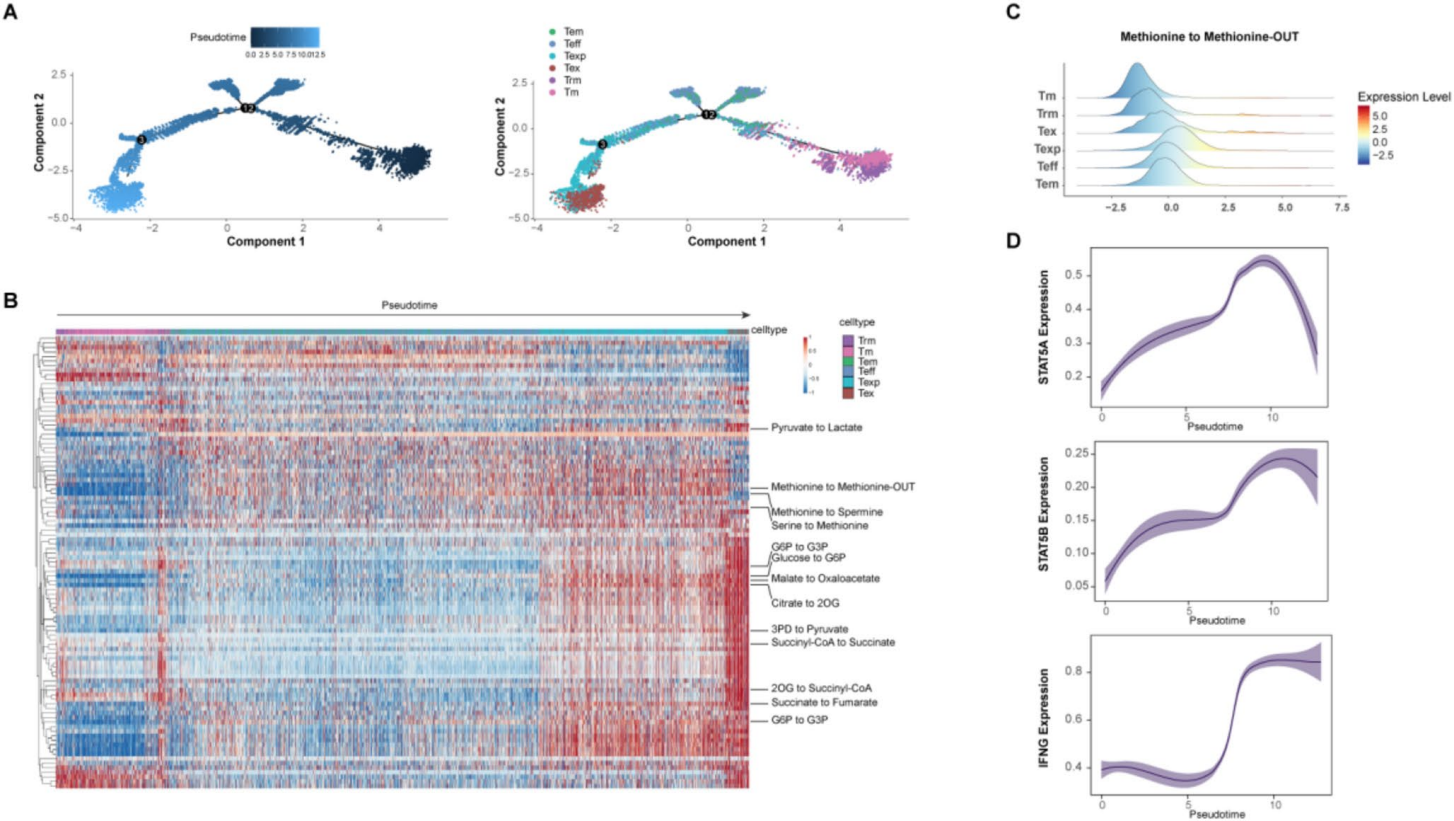
T cell remodeling after therapy. The developmental trajectory of CD8^+^ T cells inferred by Monocle 2. (Data source: GSE176022). Distribution of predicted cell-wise flux of metabolic modules. Each column represents one cell and each row represents one module. Ridgeline plots indicate the distribution values of metabolic flux in different CD8^+^ T cell groups. Each ridgeline represents the flux between two metabolites, shown on the x-axis, for different CD8^+^ T cells, shown on the y-axis. Two-dimensional plots showing the dynamic expression of STAT5A, STAT5B and IFNγ during the differentiation of CD8^+^ T cells along the pseudo-time

It is worth noting that treatment can induce the differentiation of T cells. Trajectory analysis validated that transition path went from Tm to Teff to Texp to Tex cells (Fig. 4A and Fig. S3). This observation validated the progression of CD8^+^ T cells from activation to exhaustion within the TME.

As T cells differentiate into distinct subtypes with different roles, metabolic reprogramming is essential to meet their metabolic demands and facilitate interactions with other cell types [33, 34]. To investigate the metabolic heterogeneity between these T cell subtypes, we utilized the latest computational technique known as single-cell flux estimation analysis (scFEA) [17]. Our analysis revealed that the Tex and Texp cells exhibit the highest metabolic activity in the majority of metabolic processes when contrasted with other types of T cells. This was particularly evident within the metabolic pathways for glucose and amino acids. (Fig. 4B). Additionally, we found that Texp and Tex had the highest methionine metabolism scores (Fig. 4C). Given the established link between methionine, histone methylation, and STAT5 within the context of T cells that have infiltrated tumors [35, 36], we further assessed the enrichment of interferon (IFN)-γ signaling in the scRNA-seq data. Along this trajectory, the expression of STAT5A, STAT5B, and IFN-γ increased in pseudo-time (Fig. 4D). Collectively, these results suggest that the process of T cell differentiation occurred in the neoadjuvant immunotherapy in NSCLC is accompanied by metabolic reprogramming, resulting in different subtypes of T cells with different metabolic characteristics.

### The stromal signature of TME is a predictor of immunotherapy response

We further confirmed these findings by bulk RNA-seq in a prospective cohort of 20 patients with stage III NSCLC in SYSUCC. The fundamental demographic and clinical details of the study participants were summarized in Table S2. The median age was 61.5 years (range, 50–74 years), nine patients (45%) were clinical stage IIIA at diagnosis, and 11 patients (55%) were clinical stage IIIB. Nine patients (45%) had histology of adenocarcinoma, and eleven patients (55%) had squamous cell carcinoma. After median of 2.5 cycles (range, 1–3) of neoadjuvant PD-1 inhibitor plus chemotherapy, complete resection was administered and pathologic response were assessed, twelve patients (60%) achieved MPR and eight patients (40%) achieved non-MPR.

**Fig. 5:**
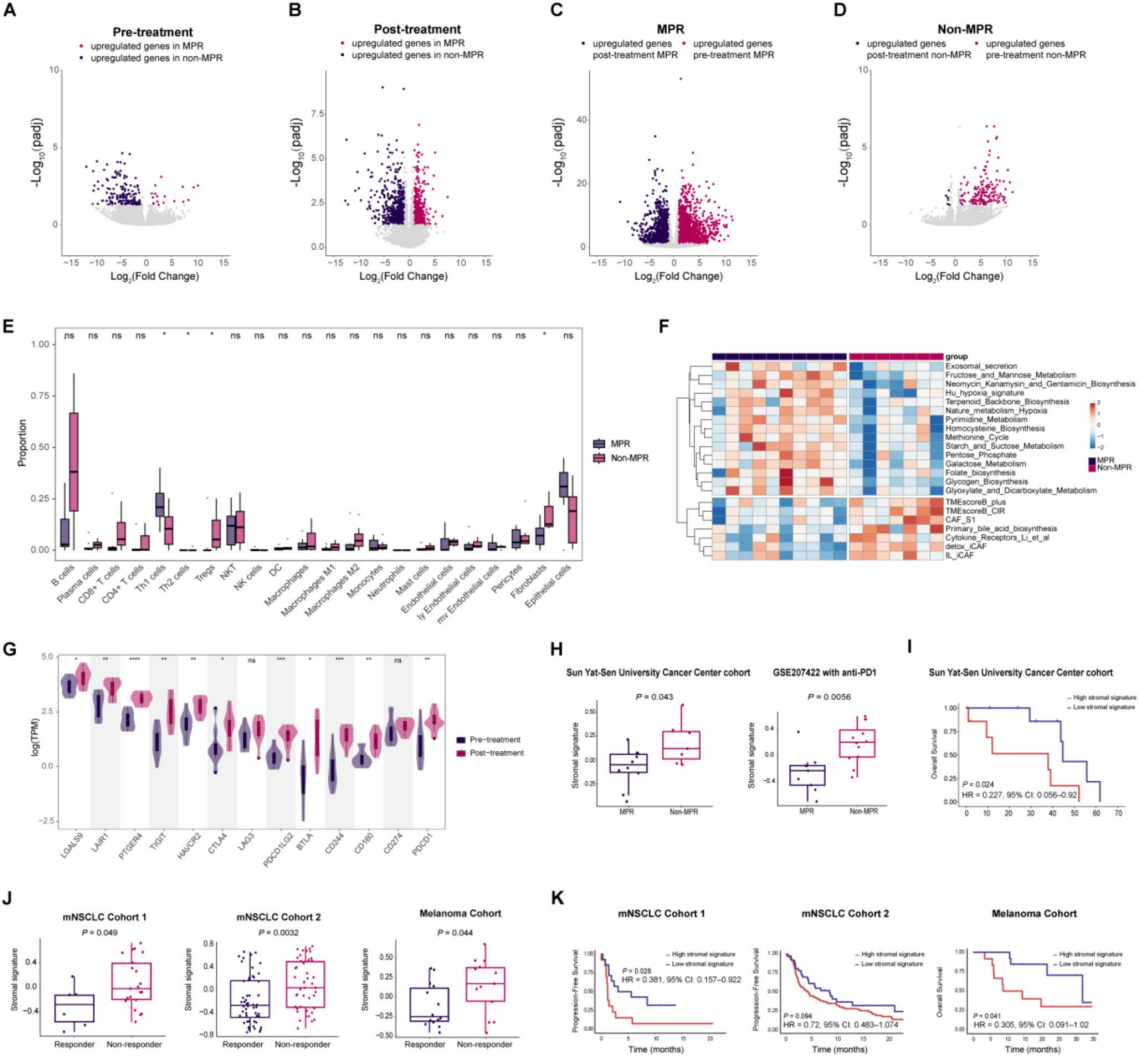
The stromal signature is a predictive biomarker of immunotherapy across multiple cohorts. (**A**) Hierarchical clustering analysis of DEGs of MPR and non-MPR patients from pre-treatment biopsies. (**B**) Hierarchical clustering analysis of DEGs of MPR and non-MPR patients from. (**C**-**D**) Clustering of DEGs of pre- and post-treatment samples from (**F**) MPR and (**G**) non-MPR patientspost-treatment tissues. (**E**) Frequency of each immune cell subtype in MPR and non-MPR patients from pre-treatment biopsies determined by xCell. (**F**) Heatmap-depicted metabolism activity and immune signatures. (**G**) Changes in checkpoint genes of MPR patients from pre- to post-treatment. (**H**) Box plots of stromal signature in two validation cohorts before neoadjuvant immunotherapy. (**I**) Kaplan-Meier survival curve of the signature of stromal in Sun Yat-Sen University Cancer Center cohort. (**J**) Box plots of stromal signature in responders and non-responders in advanced NSCLC and melanoma cohorts. (**K**) Kaplan-Meier survival curve of the signature of stromal in advanced NSCLC and melanoma cohorts. *P* value is based on non-parametric Mann-Whitney U test (**E**, **H** and **I**), and paired Wilcoxon test (**G**). Survival curves were compared by the Log-Rank test (**J**). * *P* < 0.05, ** *P* < 0.01, *** *P* < 0.001

DEGs analysis showed that 195 DEGs were identified in pre-treatment tumors between MPR and non-MPR group (Fig. 5A). In the MPR group, the upregulated genes encompassed a variety of functions, with some being part of inflammatory response pathways, such as *IL36A*, *IL36B*, *S100A9*, and *IGFL1*. Additionally, certain genes were associated with tumor antigen presentation, including *MAGEA1* and *MAGEA6*, while others played roles in metabolic regulation, with examples like *FABP5*, *SLC2A1*, *TPI1*, and *AACS*. In contrast, the non-MPR tumors exhibited upregulation of genes with a range of different functions (Table S3 and Fig. S4A-B). In post-treatment resected tumors, 405 genes were upregulated and 501 genes were downregulated in MPR group compared with non-MPR group (Fig. 5B, Table S4, and Fig. S4C-D). Upon comparing the matched samples collected before and after treatment, it was observed that a greater number of DEGs were identified in the group of patients who achieved MPR as opposed to those in the non-MPR group. This suggests a more pronounced alteration in gene expression among responders to treatment (Fig. 5C-D, Table S5-S6, and Fig. S4E-H).

The xCell algorithm was deployed to gauge the relative frequencies of various immune cell subsets within the tissue samples. MPR group demonstrated a significantly higher score of Th1 cells (*P* = 0.019) than non-MPR group, whereas non-MPR group contained a higher score of fibroblasts (*P* = 0.014) and Treg cells (*P* = 0.023) (Fig. 5E). In addition, no significant differences were noted in the expression levels of genes associated with immune checkpoints (Fig. S5A). Due to the scarcity of publicly available scRNA-seq datasets with sufficient sample size for CAF analysis in neoadjuvant immunotherapy-treated lung cancer cohorts (e.g., GSE207422 contained only 800 + CAFs), we employed a deconvolution approach using established CAF subtype-specific markers to analyze bulk RNA-seq data from our institutional cohort. This method enabled us to estimate the relative abundance of distinct CAF subtypes between MPR and non-MPR groups. Our analysis revealed a consistent trend of elevated myCAF, iCAF, and apCAF abundance in non-MPR patients compared to MPR patients, although these differences did not reach statistical significance (*p* > 0.05) (Fig. S4I). Single sample gene set enrichment analysis (ssGSEA) was also performed to characterize the tumor microenvironment (TME) in both groups, revealing that methionine cycle, nature metabolism hypoxia, galactose metabolism and glycogen biosynthesis were activated in the MPR group, while TME score and CAF gene signatures were significantly upregulated in non-MPR group (Fig. 5F). Notably, we observed strong correlations between percentage of residual viable tumor and enrichment scores of detox iCAF, TME scoreB, nature metabolism hypoxia, methionine cycle, Th1 cells, and Treg cells (Fig. S5B, *P* < 0.05). We also noticed that ECM genes were highly expressed in non-MPR tumors (Fig. S5C). These findings highlight the distinct TME compostion associated with pathological responses to neoadjuvant chemoimmunotherapy, suggesting that the TME composition plays a crucial role in treatment outcomes.

To delineate the alterations in the TME following treatment, we determined the proportions of various cellular components in both MPR and non-MPR patients. Our observations indicated an increase in the presence of T cells, dendritic cells (DCs), and B cells post-treatment in the MPR group. However, in the non-MPR group, these cell types did not exhibit a significant alteration in their proportions following the therapeutic intervention. (Fig. S6A-B). Additionally, the expression of immune checkpoint-related genes was increased after neoadjuvant immunotherapy in MPR group (Fig. 5G), underscoring that the potential of treatment to induce the activation and exhaustion of T cells in MPR tumors. Differences in the immune status of both groups based on the cancer-immunity cycle of seven stages were also investigated. In the MPR group, increased activity was observed at multiple steps in this cycle (Fig. S6C), and this process involved metabolic reprogramming (Fig. S6D).

We conducted an assessment to determine if the stromal signature derived from scRNA-seq could potentially act as a negative predictive biomarker for the efficacy of immunotherapy. The signature in question was found to score substantially higher among non-MPR patients prior to undergoing neoadjuvant immunotherapy in our study group as well as in an external NSCLC cohort (Fig. 5H). Consistent with these findings, survival analysis within our institutional cohort revealed that elevated stromal gene signature expression correlated significantly with worse clinical outcomes [log-rank *P* = 0.024, hazard ratio (HR) = 0.227; 95% confidence interval (CI): 0.056–0.92; Fig. 5I]. Subsequently, analogous analyses were conducted on three distinct publicly accessible ICB datasets, including GSE135222, the SU2C-MARK Cohort [21] and a melanoma cohort [22]. Although these cohorts were not specifically focused on neoadjuvant therapy, the stromal signature proved to be effective in forecasting responses to ICB therapy. It was observed that a higher stromal signature was present in non-responders (stable disease or progressive disease) compared to responders (complete response or partial response), with corresponding survival disadvantages (Fig. 5J and K). These findings suggest that the stromal signature can be served as a reliable biomarker for predicting poor response to ICB.

## Discussion

**Fig. 6:**
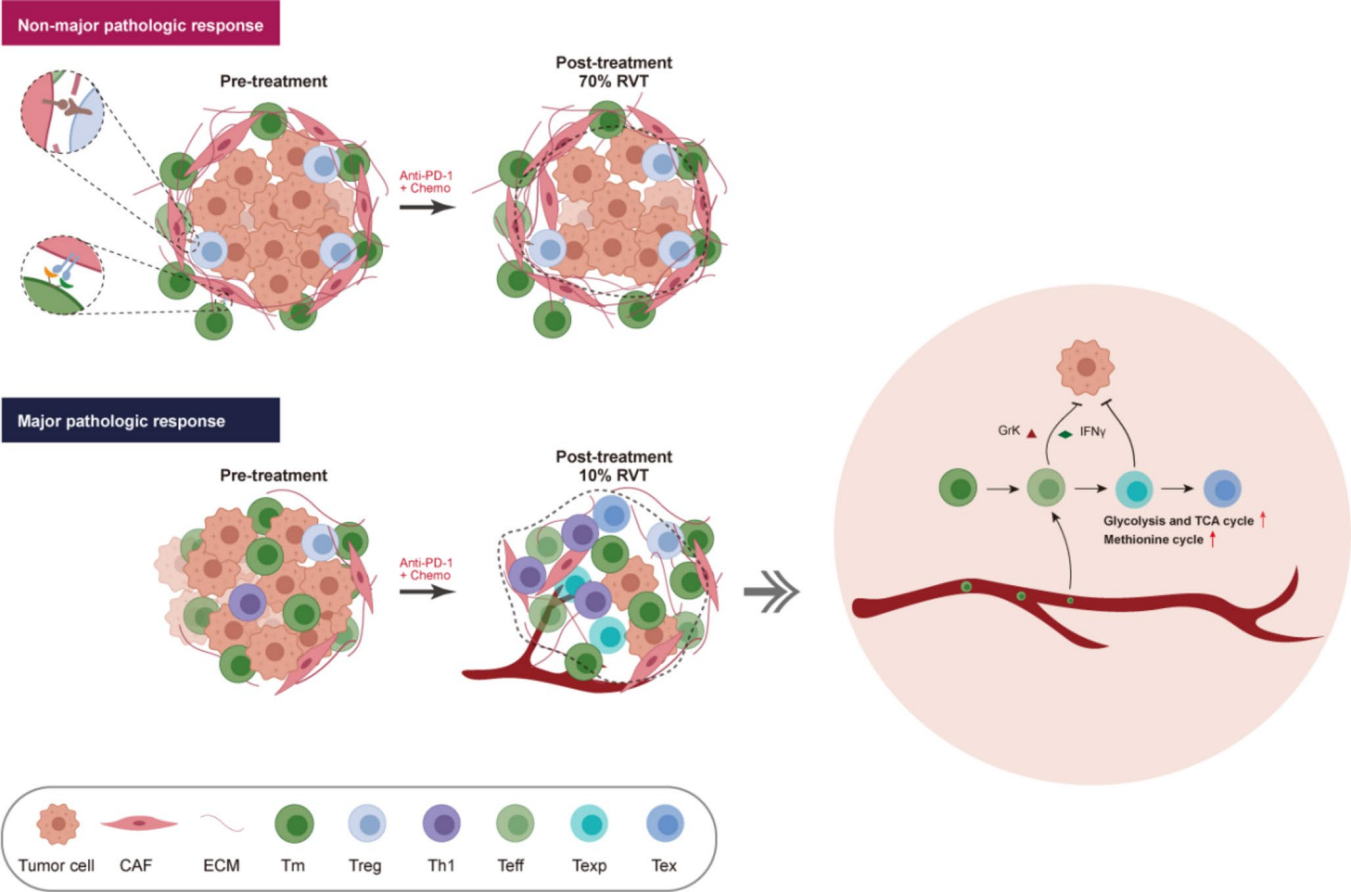
Working model. Non-MPR tumors show an enrichment of CAFs compared to MPR tumors. CAFs can interact with T cells through ligand-receptor interactions and spatially position themselves to promote the accumulation of extracellular matrix (ECM) structures, thereby excluding T lymphocytes. In MPRs, the phenotype of immune cells was remodeled after neoadjuvant immunotherapy. The memory CD8^+^ T cells were activated into an effector phenotype, including Teff and Texp cells. In addition, aberrant metabolism caused elevated energy and amino acid utilization in T cells

Locally advanced NSCLC is a group of highly heterogeneous diseases that multidisciplinary treatments should be involved in. For the population of potentially resectable patients, immunotherapy in the neoadjuvant setting have greatly improved the clinical outcome, and the group obtained MPR at surgery benefit more from neoadjuvant immunotherapy than those without MPR, which underscores the necessity for a better understanding of the determinants of antitumor immunity. Unlike in metastatic disease, neoadjuvant immunotherapy in resectable disease provides abundant on-therapy tissue samples for in-depth scientific research to explore drug mechanism-of-action and efficacy biomarkers. scRNA-seq is a potent methodology for elucidating the cellular heterogeneity within intricate biological systems, however, previous scRNA-seq analyses of neoadjuvant immunotherapy have primarily focused on post-treatment tumor samples, as obtaining high-quality paired pre- and post-treatment samples is challenging [9, 10]. In line with these efforts, we examined transcriptomes from resected locally advanced NSCLC before and after immunochemotherapy, providing a comprehensive understanding of the entire tumor microenvironment (TME) across different pathologic responses (Fig. 6).

Our findings indicated a propensity for CAFs to congregate at the periphery of the tumor, where they play a role in impeding the infiltration of T cells into the central regions of the tumor. This observation remains consistent across both advanced NSCLC and early-stage lung cancer [37], as well as in recent reports on other tumors, including breast cancer [38] and liver cancer [39], thereby revealing a potential mechanism for primary resistance to ICBs in a multiplicity of cancer entities. The compact networks of collagen fibers, secreted by CAFs within the tumor nests, have been demonstrated to form a physical barrier that hinders T cells from reaching the tumor bed [40].

In this study, we advance the understanding by demonstrating that CAFs possess the capability to alter the phenotype of immune cells in a manner dependent on immune checkpoint receptor-ligand interactions. Few studies so far have examined CAFs expressing checkpoint ligands, such as PD-L1 and PD-L2, and in this way, they impact T cell activation [41–43]. We demonstrated here that the CAFs express a variety of checkpoint ligands, with galectin-9 (encoded by *LGALS9*) being particularly noteworthy (Fig. S2). Notably, interactions between galectin-9 and its partners were enriched in crosstalk between CD8^+^ T-cell and myCAF but not iCAF (Figrue 3 C). This suggests that galectin-9 may play a role in the biology or function of CAFs. A previous study reported that the binding of galectin-9 to CD44 strengthened the immunosuppressive effects of Treg cells by stimulating the TGF-β signaling and promoting the expression and stability of Foxp3 [44]. The *LGALS9-HAVCR2* (TIM3) ligand-receptor pair has also been found to be associated with T cell apoptosis [45]. The expression of *LGALS9* in CAFs indicates a potential involvement in the tumor microenvironment through its interaction with T cells. Further analysis or experiments might be required to explore the specific role of galectin-9 in CAFs and its impact on immune modulation. Collectively, our data indicates that CAFs may play a direct role in dampening anti-tumor T-cell responses. This is achieved by promoting the demise and dysfunction of tumor-specific T cells, which in turn, leads to an increase in tumor viability. This mechanism mediated by CAFs sheds new light on the cell biology of tumor-associated fibroblasts, providing an explanation for the association of CAFs with poor prognosis in patients. Additionally, it illustrates an innovative mechanism concerning the depletion and dysfunction of T cells within the TME.

In the context of post-treatment tissue samples, our observations indicated no estimated cell changes within the paired tumor samples from patients who did not achieve MPR. This finding suggests a lack of effective immune stimulation following the treatment regimen. Conversely, MPR tumors showed upregulation of lymphocytes infiltration and gene expression profile of immune checkpoints, supporting a more proficient antitumoral immune response. It is worth mentioning that the MPR group had a higher proportion of Texp compared to the non-MPR group. Previous studies have indicated that Texp cells are likely to be the primary cellular responders to PD-1 blockade [32, 46–50]. Moreover, this Texp subset had similarities to circulating “effector-like” cells, as indicated by the expression of *STMN1* and *GZMK* (Fig. 2E). Importantly, Texp cells can undergo terminal exhaustion, and our findings suggest the potential replacement of exhausted T cells in tumors with newly converted Texp cells following PD-1 blockade. This is consistent with the phenomenon of clonal replacement that has been observed in humans after PD-1-based therapies [32, 51]. Additionally, Our findings of an increased proportion of Tem cells in non-MPR patients may reflect a dysfunctional immune activation state. While Tem cells possess cytotoxic potential [52], their accumulation without complete differentiation into effector or exhausted subsets indicates an immunosuppressive tumor microenvironment that hinders proper T cell maturation. This could result from insufficient co-stimulation (e.g., CD28/B7 axis suppression) [53], sustained inhibitory signaling (e.g., PD-1/CTLA-4) [54], or metabolic constraints (e.g., hypoxia) [55]. Importantly, T cells are increasingly recognized as existing along a continuum of functional states rather than as discrete subtypes. Transcriptomic evidence suggests that factors such as chronic stimulation and metabolic constraints dynamically shape T cell states, enabling transitions between partially activated, effector, and exhausted-like phenotypes. These findings underscore the necessity of further investigations using single-cell transcriptomics to better capture the spectrum of T cell states within the tumor microenvironment.

Our study also provided additional insights into the metabolic features in distinct T cell clusters after neoadjuvant immunotherapy in resected NSCLC. It is known that metabolic changes in tumors affect the composition and function of the non-cancer cells residing in the TME [56]. In this regard, we found that during the T-cell activation, memory T cells differentiate into effector T cells and Texp / Tex cells via transcriptional regulation, in a process supported by metabolic reprogramming to meet different energy demands. Strikingly, our data supported high methionine metabolic states in Teff / Texp / Tex versus Tm and Trm (Fig. 4C). A recent study has demonstrated the importance of methionine recycling, which serves as a donor of methyl groups in cellular methylation processes, thus enabling the epigenetic reprogramming necessary for T-cell differentiation [36]. Moreover, T-cell exhaustion driven by tumor methionine metabolism was also found in hepatocellular carcinoma [57]. In light of the direct methionine competition between tumor cells and T cells, targeted methionine restriction becomes a critical strategy for sustaining T cell-mediated immune responses in cancer patients.

While IFNs have traditionally been regarded as crucial mediators of anti-tumor immunity [58], emerging evidence reveals a more complex, context-dependent role. Beyond intrinsic tumor cell metabolic reprogramming, exogenous regulatory networks in the tumor microenvironment can synergistically amplify IFN-γ signaling cascades. Notably, seminal work by Andy Minn’s team has demonstrated that chronic IFN-γ exposure triggers tumor immunoediting programs that promote CD8^+^ T cell exhaustion and drive acquired resistance to ICB therapy [59–63]. Complementing these findings, Cao et al. recently identified a novel IFN-γ-mediated immunosuppressive axis wherein JAK1/2-STAT1-IFI6/27 signaling drives the expansion of apCAFs. These apCAFs subsequently recruit FOXP1^+^ regulatory T cells through PD-L2-RGMB interactions, contributing to neoadjuvant immunotherapy resistance in early-stage lung cancer [64]. This paradoxical duality of IFN-γ signaling - where acute activation enhances anti-tumor immunity while chronic stimulation promotes immune escape - highlights the critical importance of therapeutic timing.

Our study highlights the intricate interplay between stromal and immune components in shaping immunotherapy responses, though certain limitations merit consideration. Recent studies have increasingly highlighted the critical roles of B cells and macrophages in shaping tumor immunotherapy responses [10, 65, 66]. However, limitations in data resolution (bulk RNA-seq) and the absence of single-cell or spatially resolved datasets in our cohort precluded a deeper investigation into these populations, including their potential involvement in tertiary lymphoid structure (TLS) formation or functional polarization of macrophage subsets. Nevertheless, recent studies suggest that CAFs play a pivotal role in orchestrating immune cell crosstalk in therapy resistance [29], underscoring the need for spatially resolved multi-omics to resolve how CAF-immune cell networks (e.g., *LGALS9-TIM3* or *SPP1*-*PTGER4* axes) are spatially organized. Future studies integrating single-cell transcriptomics with spatial technologies could systematically map TLS architecture, macrophage functional transitions, and CAF subtype localization. Another key limitation of the current study design lies in its inability to delineate the distinct contributions of immunotherapy and chemotherapy to immune microenvironment modulation. While immunotherapy primarily acts by reversing immune suppression (e.g., through checkpoint blockade to reinvigorate exhausted T cells [54]), chemotherapy exhibits multifaceted immunomodulatory effects [67–70]. These include: (1) induction of immunogenic cell death to enhance tumor antigen exposure; (2) modulation of immune cell populations within the TME; (3) alteration of antigen presentation processes; and (4) reshaping of cytokine networks. Notably, emerging evidence suggests chemotherapy can directly activate NK cell-mediated tumor killing [66], adding another dimension to its immunomodulatory potential. This constraint highlights the need for future controlled studies to systematically compare these two treatment modalities. Such investigations should aim to: (1) dissect their respective mechanistic contributions to TME remodeling; (2) identify potential synergistic interactions; and (3) determine how these differential effects translate to clinical outcomes. Elucidating these aspects will be essential for optimizing combination strategies in NSCLC treatment.

In conclusion, our study provides data that CAFs and accompanied metabolic reprogramming as potential predictive factors for pathologic response in neoadjuvant immunotherapy for resected locally advanced NSCLC. In parallel, the examination of dynamic changes in the TME during treatment opens the possibility of personalized immunotherapy-based regimens in the neoadjuvant setting and even personalized multidisciplinary strategies for this highly heterogeneous disease.

## Electronic supplementary material

Below is the link to the electronic supplementary material.

Supplementary Material 1

Supplementary Material 2

## Data Availability

The data that support the findings of this study are available within the Article and its Supplementary Information or from the corresponding authors upon reasonable request.

## Abbreviations

NSCLC: Non-small cell lung cancer
MPR: Major pathologic response
scRNA-seq: Single-cell RNA sequencing
CAF: Cancer-associated fibroblasts
Texp: Precursor exhausted T
ICB: Immune checkpoint blockade
TMB: Tumor mutational burden
TME: tumor microenvironment
pCR: Pathologic complete response
RVT: Residual viable tumor
FFPE: Formalin-fixed paraffin embedded
DEG: Differential-expressed gene
UMAP: Uniform Manifold Approximation and Projection
scFEA: Single Cell Flux Estimation Analysis
RSF: Random survival forest
ssGSEA: Single sample gene set enrichment analysis
ECM: Extracellular matrix
HR: Hazard ratio
CI: Confidence interval
