# Correction to: Cancer-associated fibroblasts and metabolic reprogramming predict pathologic response to neoadjuvant PD-1 blockade in resected non-small cell lung cancer

**DOI:** 10.1007/s13402-025-01074-5

**Published:** 2025-05-23

**Authors:** Jiaqi Zhao, Maolin Liu, Chongmei Zhu, Zhuolin Li, Zuhui Liu, Dilimulati Abulizi, Siqing Liu, Xin Wang, Haoxian Yang, Xue Hou

**Affiliations:** 1 Department of Medical Oncology, State Key Laboratory of Oncology in South China, Collaborative Innovation Center for Cancer Medicine, Sun Yat-Sen University Cancer Center, No. 651, Dongfeng East Road, Guangzhou City, Guangdong Province 510060 PR China; 2 Department of Pathology, State Key Laboratory of Oncology in South China, Collaborative Innovation Center for Cancer Medicine, Sun Yat-Sen University Cancer Center, Guangzhou, PR China; 3 Guangzhou BioScript Biotechnology Co., Ltd, Guangzhou, PR China; 4 The Department of Breast Disease, The Fifth Affiliated Hospital, Sun Yat-Sen University, Zhuhai, PR China; 5 Department of Thoracic Surgery, State Key Laboratory of Oncology in South China, Collaborative Innovation Center for Cancer Medicine, Sun Yat-Sen University Cancer Center, No. 651, Dongfeng East Road, Guangzhou City, Guangdong Province 510060 PR China

**Cellular Oncology**

10.1007/s13402-025-01067-4

In this article the author’s name Haoxian Yang was incorrectly written as Haoxian X. Yang.

The original article has been corrected.

