# Supplementary material for: Cancer-associated fibroblasts and metabolic reprogramming predict pathologic response to neoadjuvant PD-1 blockade in resected non-small cell lung cancer
Source: Cell Oncol (Dordr). 2025 May 13;48(4):1105–19. doi: 10.1007/s13402-025-01067-4 (PMC12238122; doi:10.1007/s13402-025-01067-4)
Supplement: Supplementary file 1 — Supplementary Material 1 [file 13402_2025_1067_MOESM1_ESM.pdf]

Supplementary Material for "Cancer-Associated Fibroblasts and Metabolic Reprogramming Predict Pathologic Response to Neoadjuvant PD-1 Blockade in Resected Non-Small Cell Lung Cancer"

Journal: Cellular Oncology

Authors: Jiaqi Zhao[1]*, Maolin Liu[1]*, Chongmei Zhu[2]*, Zhuolin Li[3], Zuhui Liu[4], Dilimulati Abulizi[1], Siqing Liu[1], Xin Wang[5], Haoxian Yang[5#], Xue Hou[1#]

* Corresponding author:

Dr. X. Hou, Department of Medical Oncology, Sun Yat-Sen University Cancer Center; State Key Laboratory of Oncology in South China; Collaborative Innovation Center for Cancer Medicine, No. 651, Dongfeng East Road, 510060, Guangzhou City, Guangdong Province, P. R. China.

Tel: +86-13570569436; Email: houxue@sysucc.org.cn

Dr. H.X. Yang, Department of Thoracic Surgery, Sun Yat-sen University Cancer Center; State Key Laboratory of Oncology in South China, Collaborative Innovation Center for Cancer Medicine, Sun Yat-sen University Cancer Center, No. 651, Dongfeng East Road, 510060, Guangzhou City, Guangdong Province, P. R. China.

Tel: +86-13610052912; Email: yanghx@sysucc.org.cn

Supplementary Figures

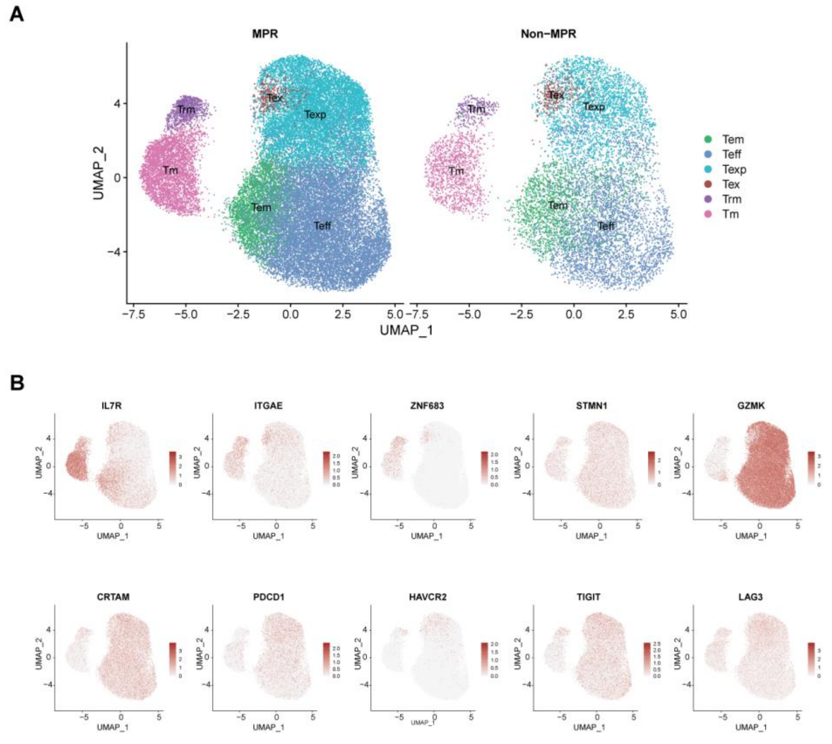

Figure S1. Identification of different clusters of CD8$^+$ T cells.

(A) UMAP plot of CD8$^+$ T cells colored by clusters in MPR and non-MPR patients.

(B) UMAP plots showing the expression levels of certain signature genes in the clusters from A.

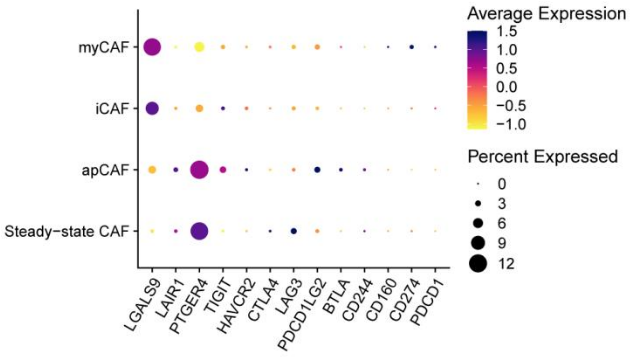

Figure S2. The expression levels of immune checkpoint-related genes in different subtypes of CAFs.

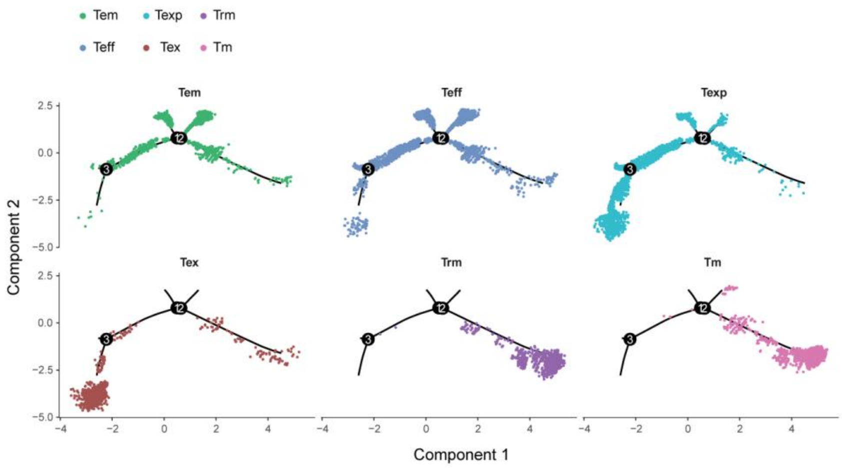

Figure S3. The developmental trajectory of $CD8^{+}$ T cells inferred by Monocle 2.

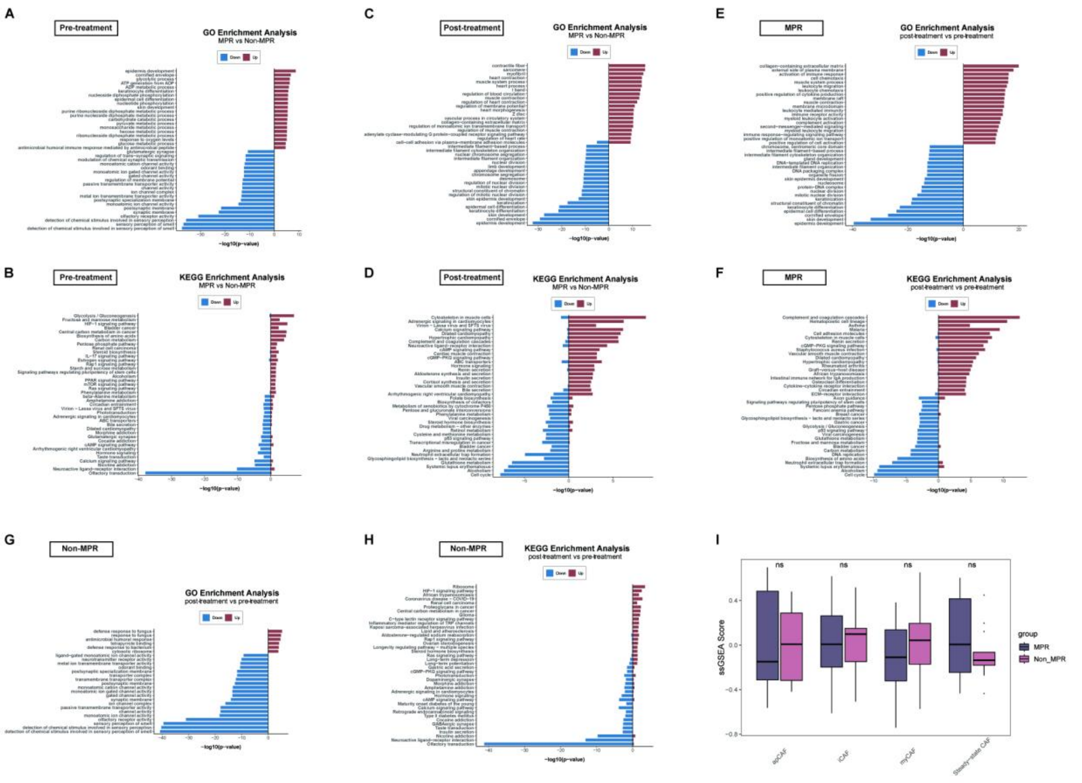

Figure S4. Pathway alterations and CAF subtype enrichment in response to neoadjuvant immunotherapy.

(A, C, E, G) GO enrichment analysis of DEGs in Figure 5A-D.

(B, D, F, H) KEGG enrichment analysis of DEGs in Figure 5A-D.

(I) Enrichment of each CAF subtype in MPR and non-MPR patients from pre-treatment biopsies determined by ssGSEA.

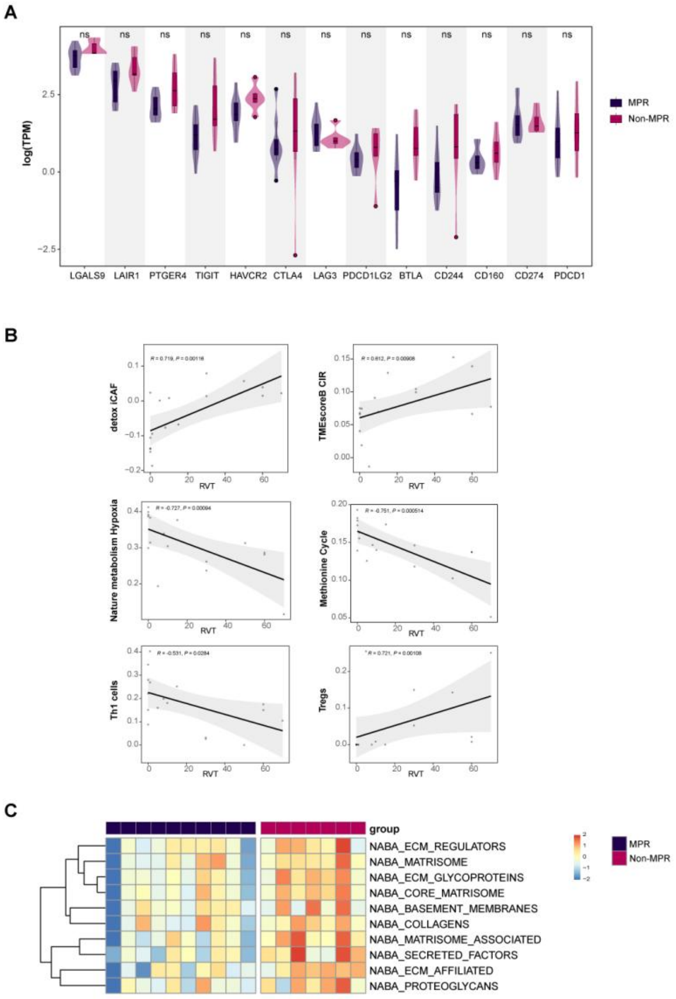

Figure S5. ECM signatures are associated with treatment resistance.

(A) Changes in checkpoint genes of MPR and non-MPR patients from pre-treatment biopsies.

(B) Estimating the correlation between the indicated signature and residual viable tumor in SYSUCC cohort. The corresponding Pearson correlation values are shown.

(C) Gene set enrichment analysis of ECM genes in MPR and non-MPR patients from pre-treatment biopsies.

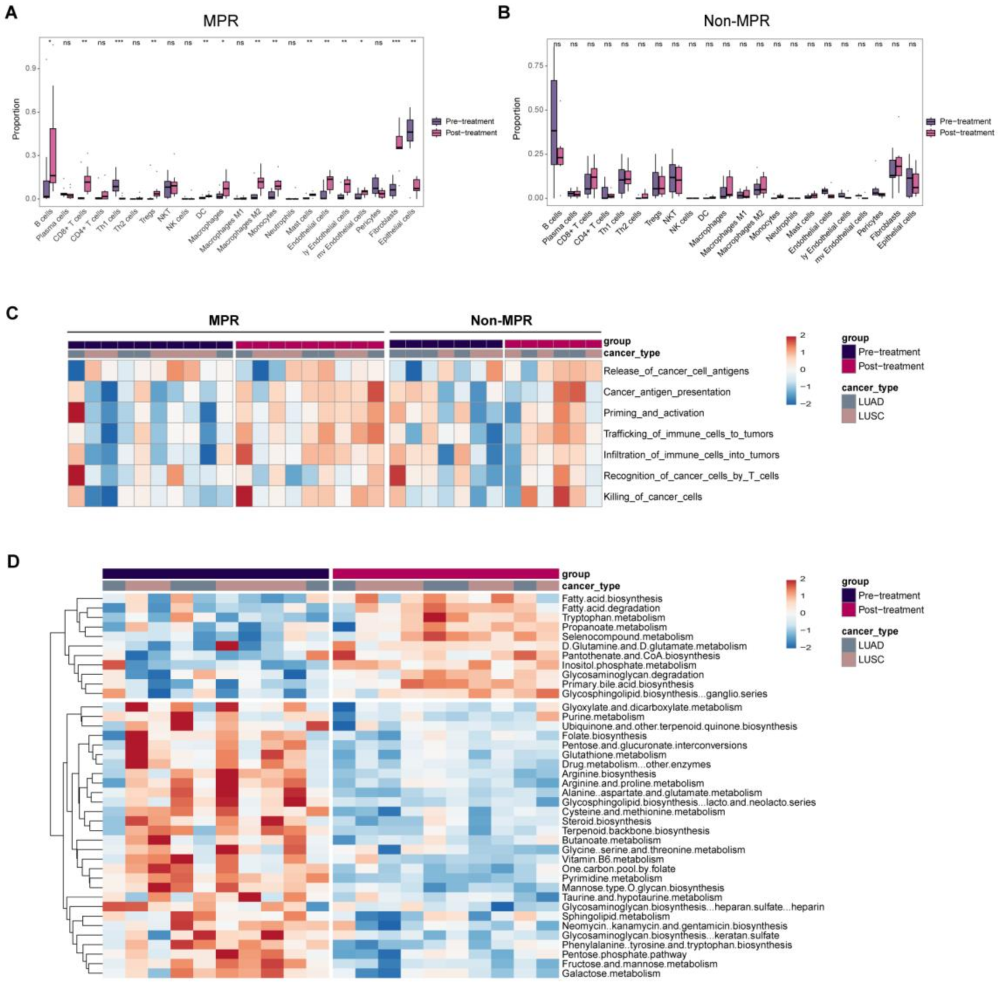

Figure S6. Anti-tumor immunity and metabolic reprogramming in MPR group.

(A) Change in immune cell populations of MPR patients from pre- to post-treatment.

(B) Change in immune cell populations of non-MPR patients from pre- to post-treatment.

(C) Heatmap for the results of TIP analysis.

(D) Metabolic pathway activities of MPR patients from pre- to post-treatment.
